# Correcting Differential Gene Expression Analysis for Cyto—Architectural Alterations in Substantia Nigra of Parkinson’s Disease Patients Reveals Known and Potential Novel Disease—Associated Genes and Pathways

**DOI:** 10.3390/cells11020198

**Published:** 2022-01-07

**Authors:** Federico Ferraro, Christina Fevga, Vincenzo Bonifati, Wim Mandemakers, Ahmed Mahfouz, Marcel Reinders

**Affiliations:** 1 Erasmus MC, Department of Clinical Genetics, University Medical Center Rotterdam, 3015 GD Rotterdam, The Netherlands; f.ferraro@erasmusmc.nl (F.F.); c.fevga@erasmusmc.nl (C.F.); v.bonifati@erasmusmc.nl (V.B.); w.mandemakers@erasmusmc.nl (W.M.); 2 Delft Bioinformatics Labaratory, Delft University of Technology, 2628 XE Delft, The Netherlands; a.mahfouz@tudelft.nl; 3 Leiden Computational Biology Center, Leiden University Medical Center, 2333 ZA Leiden, The Netherlands; 4 Department of Human Genetics, Leiden University Medical Center, 2333 ZA Leiden, The Netherlands; 5 Section Molecular Epidemiology, Department of Biomedical Data Sciences, Leiden University Medical Center, 2333 ZA Leiden, The Netherlands

**Keywords:** Parkinson’s disease, transcriptome analysis, cyto-architecture, meta-analysis, interactome analysis

## Abstract

Several studies have analyzed gene expression profiles in the substantia nigra to better understand the pathological mechanisms causing Parkinson’s disease (PD). However, the concordance between the identified gene signatures in these individual studies was generally low. This might have been caused by a change in cell type composition as loss of dopaminergic neurons in the substantia nigra pars compacta is a hallmark of PD. Through an extensive meta-analysis of nine previously published microarray studies, we demonstrated that a big proportion of the detected differentially expressed genes was indeed caused by cyto-architectural alterations due to the heterogeneity in the neurodegenerative stage and/or technical artefacts. After correcting for cell composition, we identified a common signature that deregulated the previously unreported ammonium transport, as well as known biological processes such as bioenergetic pathways, response to proteotoxic stress, and immune response. By integrating with protein interaction data, we shortlisted a set of key genes, such as *LRRK2*, *PINK1*, *PRKN,* and *FBXO7*, known to be related to PD, others with compelling evidence for their role in neurodegeneration, such as *GSK3β*, *WWOX*, and *VPC*, and novel potential players in the PD pathogenesis. Together, these data show the importance of accounting for cyto-architecture in these analyses and highlight the contribution of multiple cell types and novel processes to PD pathology, providing potential new targets for drug development.

## 1. Introduction

Parkinson’s disease (PD) is the second most common neurodegenerative disorder after Alzheimer’s disease. In PD, the loss of dopaminergic neurons in the substantia nigra pars compacta and neurodegeneration in other brain areas lead to motor and nonmotor manifestations [1,2,3]. Alpha-synuclein positive inclusions, termed Lewy bodies (LB) and Lewy neurites, are found in the surviving neurons [4]. Despite the elusiveness of the biogenesis and spreading of these structures, according to Braak’s model [5], LB pathology spreads in the PD brain along a caudo-rostral trajectory, correlating with disease progression.

Notwithstanding great progress since its initial description [6], the causative factors remain poorly understood. Various environmental, lifestyle, and genetic factors, including rare and highly penetrant variants in a limited number of genes [7] and 90 common risk loci [8], have been implicated in its pathogenesis. Nevertheless, a big proportion of missing heritability remains.

In parallel to genetic studies, genome-wide expression profiling has been used to characterize alterations in molecular pathways in different brain regions, blood, and other tissues of PD patients [9]. For example, a recent transcriptomics study reported evidence of differential brain regional vulnerability to PD progression in accordance with the Braak’s hypothesis [10]. On the basis of its role in PD, the substantia nigra has been extensively investigated in these genome-wide expression profiling studies.

Further insight into PD pathogenic pathways has been enabled by the aggregation of small-scale, low-powered, and low-concordance studies [11] into larger meta-analyses, which has led to the nomination of putative key regulators of disease progression [12,13]. This approach has proven to be fruitful, even in the context of a high degree of heterogeneity in the putative causes and severity of phenotypes of the included patients.

Unfortunately, transcriptomic studies suffer from various technical limitations, such as RNA degradation, which affect the different cell types to a variable extent [14]. Furthermore, differences in cyto-architecture originating from biological heterogeneity (due to, e.g., age, gender, and genetic background), as well as sample preparation, can further influence downstream analyses. An even stronger confounder might be represented by cell composition changes induced by a pathology [15]. This issue is particularly concerning since it is not possible to distinguish between changes in genes that are highly expressed in a cell type whose proportions change and a genuine pathology-related transcriptional deregulation. While the former might be interesting as it may correlate with disease progression, the latter can inform on the molecular mechanism for which therapeutic strategies might be devised. In neurodegenerative disorders and especially in highly affected brain regions, such as the substantia nigra in PD, this phenomenon might be even more pronounced.

To tackle these and other challenges, single-cell RNA sequencing (scRNAseq) and single-nucleus RNA sequencing (snRNAseq) are currently rising in popularity. However, thus far, only a limited number of PD midbrain samples have been profiled at the single-cell level [16], severely decreasing the power to detect relevant differences. Alternatively, cell proportions can be estimated from bulk transcriptomics data, and then analyses can be corrected for altered cyto-architecture. Recently, several bioinformatic approaches have been proposed to estimate and use these surrogates for the proportions of the cell types, offering the opportunity to exploit the enormous amount of data readily available in public repositories [17,18,19,20,21,22,23,24]. So far only a few attempts have been made in this direction for PD substantia nigra. These are based on simply correlating gene expression and neurodegeneration across brain regions and are either limited to the dopaminergic neurons [25] or to a limited number of substantia nigra samples [10].

**Figure 1:**
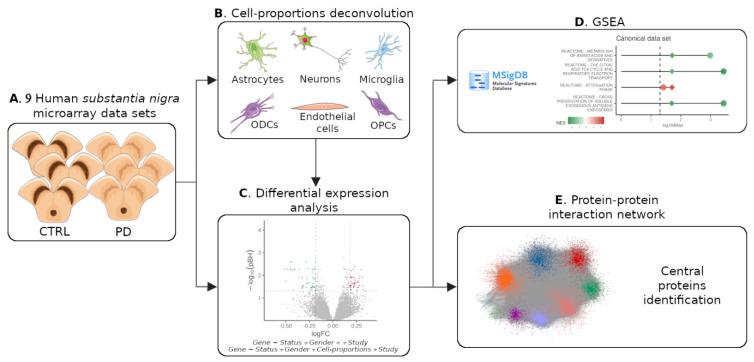
Summary of this study. (**A**) Human substantia nigra microarrays from PD and CTRL were downloaded from GEO. (**B**) Cell-proportion deconvolution for six cell types. (**C**) Differential expression analysis with and without cell proportions. (**D**) GSEA on the expression matrix. (**E**) Central protein identification in PPI network around detected differentially expressed genes. Drawings of substantia nigra and cells were obtained from Servier Medical Art templates (Creative Commons Attribution 3.0 Unported License; https://smart.servier.com accessed on 1 May 2021).

In this study, we systematically assessed the transcriptomic evidence for the presence of changes in cell compositions in the expression data of nine publicly available Parkinson’s disease-related microarrays of the substantia nigra (see Figure 1 for an overview). We conducted the first meta-analysis of these datasets while evaluating the impact of the cyto-architectural alterations on the differential expression analysis. By correcting for these effects, we were able to detect genuine disease-related changes in the transcriptional landscape of the substantia nigra in PD patients. Lastly, we explored the protein interactome of the identified deregulated genes and nominated promising candidates for further investigations by exploiting their network characteristics.

## 2. Materials and Methods

### 2.1. Transcriptome Dataset Acquisition, Preprocessing, and Gender Imputation

We downloaded human gene expression datasets from the Gene Expression Omnibus (GEO) using “Parkinson’s disease” and “substantia nigra” as keywords. In total, we found 10 studies; nine (GSE7621, GSE20333, GSE20292, GSE20163, GSE20164, GSE54282, GSE49036, GSE43490, and GSE42966) analyzed the substantia nigra for each patient, while one separately analyzed the medial and the lateral part of the substantia nigra (GSE8397), from which we only used the lateral part as it is more affected in PD [26]. GSE54282 was excluded from analysis because of the small number of available samples. Only probes mapping to an Entrez ID using the biomaRt package version 2.42.1 [27] were kept. When multiple probes mapped to the same Entrez ID, we kept the one with the maximum variance and connectivity using the collapseRows function from the WGCNA package version 1.69 [28], resulting in a total of 18,948 genes. We used gene expression as already processed in the original studies (either Robust Multichip Average normalization or the Affymetrix microarray suite), and we removed outlier samples according to the original publications. Finally, expression data were log-transformed, and the merged dataset was quantile normalized with the normalizeBetweenArrays function from the limma package version 3.42.2 [29]. Since gender information was not always available, we used the massiR package version 1.22.0 [30] to annotate all the samples using the top 75% variable genes for the prediction.

### 2.2. DEGs Meta-Analysis

**Figure 2:**
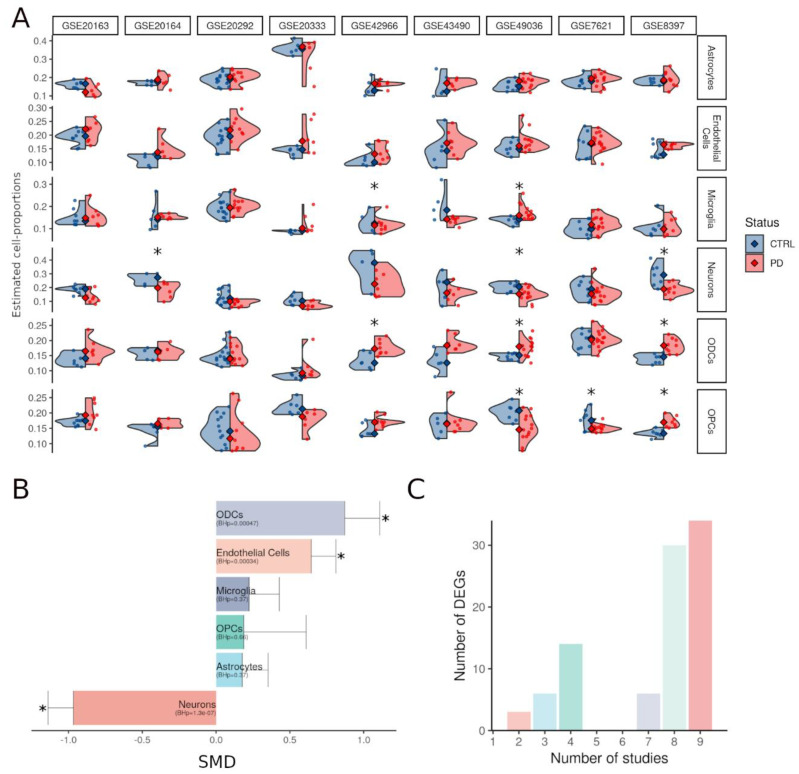
Cyto-architectural heterogeneity as estimated from bulk transcriptomics using the deconvolution strategy of TOAST. (**A**) Cell estimates of six cell types in substantia nigra across different datasets and conditions. Blue, CTRL; red, PD. Significant variations are indicated by asterisk (*p* < 0.05). (**B**) Bar plot of the standardized mean differences (SMD) in cell estimates from the random-effects meta-analysis conducted with metafor. BH-adjusted *p*-values are reported between brackets, significant differences are indicated by asterisks “*” (BH *p* < 0.05), and standard errors for each cell type are reported as error bars. (**C**) Number of DEGs of the cell proportion-aware LMM probed by a specific number of microarrays in our dataset.

The DEGs were identified by fitting a linear mixed-effects model (LMM). For each gene probed by two or more studies, two LMMs were fitted using the lmer function from the lmerTest package version 3.1.2. One LMM accounts for the status and the gender as fixed effects and the different studies as random effects: *Gene_exp_ ~ Status + Gender + (1|Study)*, with *(1|Study)* indicating a one-hot encoding of the study. The other LMM also accounts for estimates of cell types (neurons (NEU) and oligodendrocytes (ODC)) as fixed effects: *Gene_exp_ ~ Status + NEU + ODC + Gender + (1|Study)*. We chose to only use a subset of the cell types as covariates to avoid the introduction of collinear predictors. Specifically, we included those cell types whose estimates are only weakly correlated with the estimates of the other cell types in the model but highly correlated with the excluded ones (Supplementary Figure S1A). Moreover, we ensured to include cell types whose estimates showed changes in opposite directions between PD and CTRL (Figure 2B). The *p*-values were corrected using the Benjamini-Hochberg (BH) method.

### 2.3. Processing of Substantia Nigra Single-Nucleus Data

Human substantia nigra single-nucleus RNAseq data [31] were downloaded from GEO (GSE140231) and preprocessed with Seurat package version 2.3.0 [32] according to the original publication. Cell markers for each cluster compared to all the others were identified from transcripts detected in at least 30% of the available cells, with a log fold change higher than 0.5 using the function FindAllMarkers. The clusters were broadly annotated into six cell types using well-known markers: *GFAP* and *GINS3* for the astrocytes; *MOBP* and *MOG* for the oligodendrocytes; *CSF1R* and *OLR1* for the microglia; *GAD1*, *GAD2*, *GABRA*, SLC6A3, and *TH* for the neurons; *VCAN* for the oligodendrocyte precursor cells; *LGALS1* and *RGS5* for the endothelial cells.

### 2.4. Cell-Type Proportion Estimation and Marker Selection

Cell-type proportions were estimated by deconvolution using the function MDeconv from the TOAST package version 1.1.2 [24,33]. We considered six brain cell types: astrocytes, endothelial cells, general neurons, microglia, ODCs, and OPCs. We also repeated the deconvolution step and estimated the proportions of the same cell types, as well as of dopaminergic neurons in place of the general neuronal population. We used *TH* and *SLC6A3* as input markers to infer the dopaminergic neuron proportion. For the other six cell types, we used subsets of 20 markers for each study selected among the 5500 markers from the Brain Cell Type—Specific Gene Expression Analysis package version 1.0.0 (BRETIGEA) [19] (Supplementary Materials). Correlation analysis of the BRETIGEA markers shows clustering by cell types (Supplementary Figure S2). Furthermore, we evaluated whether the BRETIGEA-derived marker sets were enriched in the correct substantia nigra cell type using expression-weighted cell-type enrichment (EWCE) (Methods) (Supplementary Table S1). This showed that, in 50 of the six cell types for each of the nine studied cases (93%), the markers were enriched in the expected cell types (Supplementary Table S1). The four cases in which the sole enrichment was not in the expected cell types all involved OPC markers. Two studies (GSE43490, GSE7621) showed a close-to-significant enrichment after the BH correction; in one study (GSE49036), the OPC markers were significantly enriched in the expression signature of OPCs and neurons, while, in the other study (GSE20292), no significant enrichment was found in any cell type for the OPC markers.

### 2.5. Cell-Type Proportion Comparison and Statistical Analyses

For each study and cell type, statistically significant differences in the estimates of a cell type proportion between the CTRL and the PD substantia nigra were assessed with a linear model controlling for the other annotated covariates, i.e., *Proportion_CellType_ ~ Status + Gender + Age + BraakStaging*. To verify the shared alterations in proportions across the studies, we also conducted a random-effects meta-analysis with metafor version 2.1.0 [34]. For each deconvoluted cell type and study, the standardized mean difference was calculated between cases and controls. Effect sizes were combined with the rma.uni function from the metafor package. Finally, the *p*-values were corrected using the BH method.

### 2.6. Gene Set Enrichment Analysis

Gene set enrichment analysis (GSEA) was performed using the Fast gene set enrichment analysis package version 1.12.0 (fgsea) [35], 100,000 permutations, with the Gene Ontology (GO) and the Canonical dataset downloaded from the Molecular Signatures Database (MSigDB). For the GSEA on the gene expression, the genes in our dataset were first ranked in descending order by the negative logarithm in base 10 of the adjusted *p*-values multiplied for the sign of the effect size. For the GSEA of the PPI network, nodes were ranked by the betweenness centrality [36]. All *p*-values were corrected using the BH method.

### 2.7. Expression-Weighted Cell-Type Enrichment (EWCE)

Gene set enrichment for specific cell types was done using the expression-weighted cell-type enrichment (EWCE) [37] package version 1.0.1. To this aim, we first calculated the average expression matrix for each cell type in the substantia nigra using the AverageExpression from the Seurat package using the GSE140231 dataset. For each tested list, 10,000 randomly sampled (equal sized) gene sets from all genes in the average expression matrix were used to calculate the *p*-values, which were then adjusted using BH correction.

### 2.8. Protein-Protein Interaction Network Construction and Analysis

A protein-protein interaction network was built from six publicly available databases: (1) the Human Reference Interactome and Literature Benchmark (HuRI) [38], (2) the Biological General Repository for Interaction Datasets (BioGRID build 3.5.186) [39], (3) STRING v11 database [40], (4) the Integrated Interactions Database, Version 2018-11 (IID) [41], (5) BioPlex 3.0 [42], and (6) IntAct Database [43]. For all databases, only experimentally validated interactions were selected, the lists were merged, and nonhuman proteins, nodes with just one edge, self-interactions, and duplicated interactions were removed. The final graph was visualized in Cytoscape 3.8.0 and analyzed with the R package igraph 1.2.5 [44]. Fisher’s exact test (FET) was used to test for the enrichment in the whole network and in the central proteins for typical PD-causing genes, selected according to MDSGene (https://www.mdsgene.org/g4d (accessed on 4 April 2021)), and for the genes in proximity of PD risk variants [8] (downloaded from GWAS Catalog, https://www.ebi.ac.uk/gwas/ (accessed on 4 April 2021)). The number of human protein-coding genes was used as background for the tests on the whole network, while the network size was used for those with central proteins.

## 3. Results

### 3.1. Alterations in Composition of the Substantia Nigra Are Heterogeneous

In total, 70 control (CTRL) and 88 PD transcriptomes were put together in our study. After imputing the gender for all samples (Methods), there were 62 females (27 CTRL, 35 PD) and 96 males (43 CTRL, 53 PD), with an average age of death of 75 years (Table 1). To capture the PD induced cyto-architectural alterations, we estimated the proportions of six cell types (astrocytes, endothelial cells, general or dopaminergic neurons, microglia, ODCs, and OPCs) from the bulk data using computational deconvolution (Methods). This revealed cell proportion heterogeneity; not only between the two groups, i.e., CTRL and PD patients, but also within each group (across samples), as well as between datasets (Figure 2A, Supplementary Table S2). Although we observed similar trends between CTRL and PD brains, the alterations in cell proportions were not consistently replicated across the analyzed datasets. To identify consistent alterations, shared among datasets, we conducted a meta-analysis of the estimated cell type proportions. This showed a significant increase in endothelial cells and oligodendrocytes as opposed to a decrease in the neuronal estimates (Figure 2B, Supplementary Table S2). In the meta-analysis, the change in the fraction of neurons was the strongest, followed by that in ODCs and then in endothelial cells. The estimated fraction of dopaminergic neurons was highly concordant with that of the general neuron population (average Pearson’s correlation coefficient 0.81 ± 0.16), suggesting that we can use the fraction of neurons as proxy for that of dopaminergic neurons. This is beneficial as the fraction of neurons can be estimated using a larger number of markers, thus resulting in more accurate estimates.

### 3.2. The Cyto-Architectural Alterations Are a Major Confounder in the DEG Identification

To detect consistent differentially expressed genes (DEGs), we fitted a linear mixed-effects model (LMM) for each gene across all datasets, with a random effect for the different studies and fixed effects for gender and CTRL/PD status (Methods). Out of the 1050 deregulated genes between PD and control samples (adjusted *p*-value < 0.05, fold change (FC) > 1.2), 702 were downregulated and 348 were upregulated (Supplementary Table S3). We then included estimates of the neurons and ODCs as fixed effects into the linear mixed effects model (Methods). We chose to correct only for these two cell types due to their collinearity with the other cell types (Supplementary Figure S1A). With the second LMM, we detected a reduced number of DEGs (adjusted *p*-value < 0.05, FC > 1.2): 93 instead of the 1050 obtained with the cell proportion-unaware LMM (63 genes overlapping). Out of the 93, 50 were downregulated and 43 were upregulated in PD with respect to the CTRL samples (Supplementary Table S3, Supplementary Figure S1B). The majority of the DEGs (69%) were probed in more than seven studies, showing a robust alteration signature across the datasets despite possible heterogeneity in etiological factors and pathology severity (Figure 2C). None of the PD-causing genes nor any of the genes proximal to GWAS risk variants were found among the DEGs. To find out for which cell types the 93 DEGs were enriched, we performed an expression-weighted cell-type enrichment with EWCE (Methods). Downregulated genes appeared to be significantly enriched in neurons, whereas upregulated genes were not enriched in a specific cell type (Supplementary Table S4). No significant enrichment was found for the other cell types. 

### 3.3. GSEA Reveals That the Majority of the Altered Pathways in PD Are Downregulated

**Figure 3:**
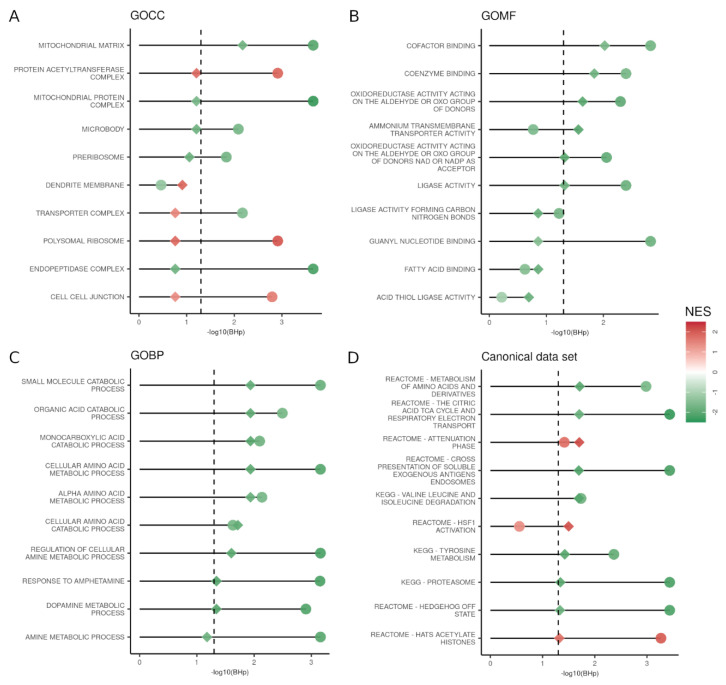
Comparison of the GSEA for the genes ranked by the two linear mixed models (LMMs). Top 10 significant hits of the cell proportion-aware (second) LMM (diamond) compared with their respective values from the cell proportion-unaware (first) LMM (circle). Each shape is colored by the normalized enriched scores (NES); −log_10_ of the adjusted *p*-value is reported on the *x*-axis. The significance threshold (BHp = 0.05) is indicated by the dashed vertical line. (**A**) Top results for gene ontology cellular component (GOCC); (**B**) Top results for gene ontology molecular function (GOMF); (**C**) Top results for gene ontology biological process (GOBP); (**D**) Top results for canonical data set.

To explore the effect of the PD-related gene expression deregulation, we conducted a gene set enrichment analysis (GSEA) for functions in GO and the MSigDB canonical data set (Methods). Genes were ranked by the signed corrected −log_10_ *p*-values obtained from either LMM. Similarly, to the DEG analyses, accounting for cell composition decreased the number of significant pathways (adjusted *p*-value < 0.05) (Figure 3, Supplementary Table S5). Specifically, 253 significant canonical pathways (92 upregulated and 161 downregulated) were identified with the cell proportion-unaware (first) LMM. When applied to the expression matrix ranked by the cell proportion-aware (second) LMM, only 12 pathways (two upregulated and 10 downregulated) remained significant, and one pathway, upregulation of the heat-shock transcription factor 1 (*HSF1*) activation pathway, became significant. Among the pathways that lost significance between the two LMMs, there were pathways such as neurotransmitter receptors and postsynaptic signal transmission (Reactome), Parkinson’s disease (KEGG), oxidative phosphorylation (KEGG), and alpha-synuclein (PID) pathways.

### 3.4. PPI Partners of DEGs Are Enriched for Genes Expressed in Endothelial Cells and Neurons

**Figure 4:**
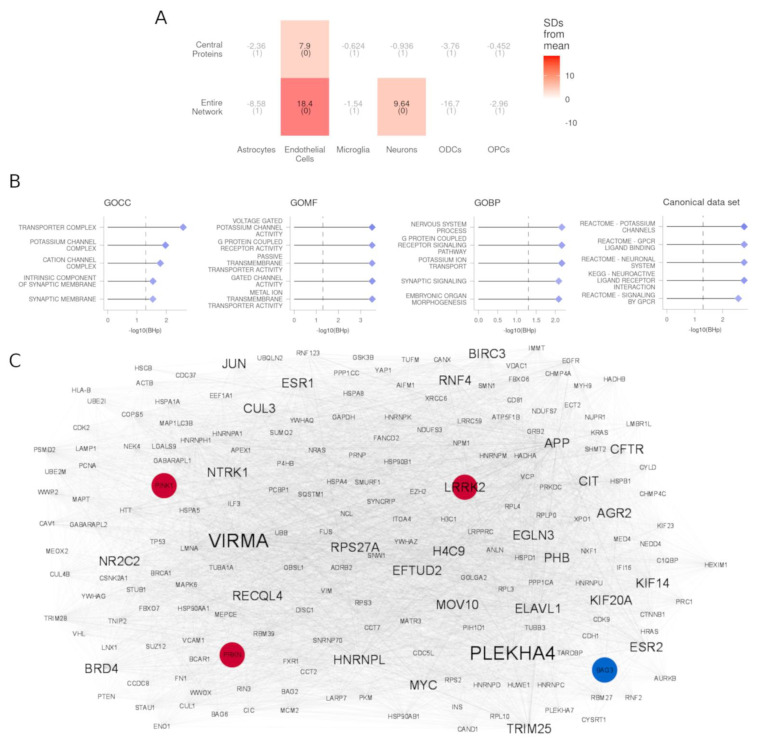
PPI network analyses. (**A**) EWCE results for the full network and central protein. The number of standard deviations of the expression of each list from the mean level expression of 10,000 equally sized random lists is reported for each cell type. BH-corrected *p*-values are reported between brackets. (**B**) GSEA results of the nodes in the network ranked by betweenness centrality. The −log_10_ value of the adjusted *p*-value is reported on the *x*-axis, and the significance threshold (BHp = 0.05) is indicated by the dashed vertical line. (**C**) Network of the central proteins extracted from the full PPI network. Label size is proportional to the protein degree, edge thickness, and color proportional to the edge betweenness. PD-causing genes (*LRRK2*, *PINK1*, *PRKN*) and the genes closest to a risk factor (*BAG3*) are highlighted in brick red and cerulean blue, respectively.

To identify the interaction partners of the proteins encoded by the DEGs, we constructed a protein-protein interaction (PPI) network based on the BioPlex, Biogrid, HuRI, IID, IntAct, and String databases (Methods). Selecting the direct interactors of the 93 DEGs, we obtained a network comprising 5705 proteins and 155,937 edges (Methods, Supplementary Table S6). We assessed the expression-weighted cell-type enrichment with EWCE of the entire network and found a significant enrichment for endothelial cells and neurons (Figure 4A, Supplementary Table S7). Among the enriched pathways (using GSEA), there were potassium channels, GABA receptor activation, SLC-mediated transmembrane transport, neuronal system, and matrisome (Figure 4B, Supplementary Table S8). Moreover, this network contained all of the six PD-causing genes.

### 3.5. Central Proteins of DEG PPI Partners Are Known PD-Causing Genes and Genes in Proximity to PD Risk Factors

Within the PPI network of DEG interaction partners, we identified 207 nodes that have both a degree centrality higher than the 95th percentile (288 nodes) and a betweenness centrality higher than the 95th percentile (288 nodes). Among these, five (2%) were encoded by DEGs. The remaining 202 central nodes were defined as top central proteins (Figure 4C, Supplementary Table S6). EWCE analysis revealed that these genes are enriched in endothelial cells (Figure 4A, Supplementary Table S7). The top proteins were also enriched in PD-causing genes (three genes out of six, FET *p*-value 8.1 × 10^−4^) and included leucine-rich repeat kinase 2 (*LRRK2*), Parkin RBR E3 ubiquitin protein ligase (*PRKN*), and PTEN-induced putative kinase 1 (*PINK1*). Moreover, two of the genes reported in proximity to GWAS risk factors were also among the central proteins: *LRRK2* and BAG cochaperone 3 (*BAG3*).

## 4. Discussion

We analyzed the influence of cyto-architectural alterations on the transcriptomic signals from human substantia nigra microarray data from PD patients and CTRL. We demonstrated that a broad palette of alterations in cell composition was present within and between strata, as well as across studies. Specifically, the significant decrease in neurons and increase in ODCs in PD with respect to CTRL were the most consistent, but differences for microglia and OPCs were also found. The lack of a universal alteration pattern might be attributed to the mixed cohort of patients assessed, with variable putative etiological factors, phenotype, and disease severity that characterize PD, as well as technical variability. Nonetheless, a meta-analysis of cell proportions showed a significant decrease in the neurons and an increase in ODCs and endothelial cells in PD. This pervasive heterogeneity heavily influenced differential expression analysis between PD patients and controls. When not correcting for cell-type composition we found 1050 DEGs in a meta-analysis. After adjusting for cell type composition, only 63 DEGs remained, along with 30 new DEGs. Together, these findings provided evidence that the systematic integration of microarrays of substantia nigra in PD, albeit a popular methodology to increase power in detecting the DEGs, resulted in many spurious associations when not controlling for cyto-architecture. The downregulated DEGs identified with the model with correction for cyto-architecture were significantly enriched in neurons, whereas the upregulated genes were not enriched in a specific cell type. This might suggest that, despite dopaminergic neurons being the most affected in PD, alterations in other cell types participate in the neurodegenerative process, in accordance with the mounting evidence in this direction [31,54,55].

Estimated cell-type proportions were supported by previous and independent studies confirming the reliability of the computational deconvolution strategy. Firstly, the observed loss of dopaminergic neurons in substantia nigra is a pathological hallmark of PD. Secondly, our observation that endothelial cells were increased in the substantia nigra of PD patients is in line with previous reports [56,57], although it is still unclear whether endothelial cell expansion is a result or a driver of the inflammation status. Indeed, angiogenesis can be stimulated by molecules secreted by astrocytes and microglia in the reactive status [58,59] in a vicious loop [60]. Thirdly, the increase in microglia in one of the studies can be related to reactive gliosis present in PD that is known to implicate this cell type [61]. Fourthly, the observed increase in OPCs and ODCs probably reflects the skewed neuron/oligodendrocyte ratio in the dissected samples due to the neuronal death. Lastly, the absence of astrogliosis is in concordance with reports showing absence or at most a mild increase in immunoreactivity for astrocytic markers in the substantia nigra of PD patients [62].

The deconvoluted cellular proportions not only uncovered interesting features of the cyto-architecture of the substantia nigra in PD and adjusted the DEG detection, but also influenced the pathway analysis. We confirmed alterations in known pathways and functions like the tricarboxylic acid cycle [63], dopamine metabolism [64], proteasome activity [65], HSF1 activation and attenuation [66], and the expression of genes activated in the hedgehog pathway in the off status [67]. Furthermore, we also identified an intriguing decrease in the ammonium transport proteins which, despite having been suggested for other pathologies [68], has not yet been reported for PD. Intriguingly, previous data suggest that ammonia accumulation could affect energy metabolism, mitochondria, inflammation, and neurotransmission [69,70]. Importantly, accounting for the cyto-architecture also showed that several of the usually reported pathways (e.g., Parkinson’s disease pathway, oxidative phosphorylation, alpha-synuclein pathway) might be driven, at least in part, by changes in cell composition rather than the pathological status. A similar observation was also recently reported in PD prefrontal cortex [71], reinforcing the contention that cyto-architecture is an important covariate with a major impact on our ability to understand transcriptional changes in bulk transcriptomics.

As interacting partners of DEGs can have consequent altered functionality, we constructed a network of protein-protein interactors with the detected DEGs. Cell type enrichment analysis of this network corroborated that gene deregulation might also have an impact on neuronal biology through the interacting partners of the DEGs. Furthermore, it showed ramifications in endothelial cell processes unidentified by the differential expression analysis. Similarly, the GSEA reinforced the involvement of the neurons and pinpointed previously undetected extracellular matrix alterations also reported to be affected in PD [72]. Importantly, PD-causing genes and those proximal to GWAS variants, albeit not being differentially expressed, were enriched in this network. This convergent evidence shows how genetically identified genes can have an impact on the transcriptional landscape of the substantia nigra even when not differentially expressed and supports the relevance of this network. Exploiting their topological characteristics, we nominated key proteins that were central in this partner network. Some of the central proteins have indeed been implicated in the pathogenesis of PD by independent lines of research, such as genes whose variants are causative of inherited forms of PD (*LRRK2, PINK1, PRKN*) [73,74,75], are causative of parkinsonian-pyramidal syndrome (*FBXO7*) [76], and/or are the closest to risk variants for developing PD (*BAG3, LRRK2*) [8]. Furthermore, other hits with compelling evidence supporting their role in neurodegeneration (e.g., *GSK3β, WWOX*, and VPC) [77,78], corroborate that new potential players in the PD pathogenesis might be identified among the central proteins we found.

Our cell-type composition-aware meta-analysis comes with some assumptions and limitations. First, we assumed that the markers used for the cell proportion deconvolution were not differentially expressed among the conditions. Secondly, by selecting the BRETIGEA markers, we assumed that cortically identified markers could be representative of the substantia nigra cell populations. Joint clustering of the substantia nigra and cortical cells by type instead of by region is, however, reported [31]. Moreover, we showed that the selected markers are enriched in the correct cell types. Together, these data suggest that this assumption can be made.

There are three major caveats to our study. Firstly, the deconvolution step does not take into account the differences in cell size or in RNA content of the various cell types, potentially leading to systematic errors [22]. Secondly, as the sum of the estimates of the cell-type proportions is constrained to be 100% for each sample, an increase or decrease in any of the cell types has to result in an equal but opposite alteration in at least one of the other cell types. Thirdly, the incomplete annotation of the samples across the studies prevented us from exploring the effect of age, age of onset, pathology progression, genotype, and Braak staging on the cell proportions and transcriptional processes, all known to influence PD severity [79,80,81].

The future advent of scRNAseq/snRNAseq studies of the human substantia nigra of PD patients will allow additional steps forward in this area of research to confirm the cyto-architectural and transcriptomics alterations. Furthermore, genetics and functional studies are warranted to illuminate the role of the central proteins we identified in PD pathological mechanisms.

In conclusion, our meta-analysis of bulk transcriptomics gives an updated view of the transcriptional landscape in the substantia nigra of PD patients. In addition to leveraging a large number of studies and samples similarly to previous works [12,13], we accounted for the dramatic cyto-architectural alteration induced by PD in this brain area, uncovering its effects on downstream analyses. Using multiple complimentary approaches, encompassing the transcriptional and protein-interaction perspectives, we implicate the multiple cell types affected in PD substantia nigra. Moreover, a number of novel PD candidates and identified enriched biological processes offer clues for a better understanding of the complex PD pathology, providing steppingstones for further research and the identification of new therapeutic approaches.

## Figures and Tables

**Table 1:** Overview of the studies contributing data to our analyses.

GEO ID	Tissue	#CTRL (F:M)	#PD (F:M)	Platform	#*n* Probes	F:M	Average Age of Death	Reference
GSE7621	SN	9 (5:4)	16 (3:13)	GPL570	54,675	8:17	78	[45]
GSE8397	LatSN	7 (2:5)	9 (4:5)	GPL96	22,283	6:10	76	[46]
GSE20333	SN	6 (1:5)	6 (5:1)	GPL201	8793	6:6	78	[47]
GSE20292	SN	15 (5:10)	11 6:5)	GPL96	22,283	11:15	73	[48]
GSE20163	SN	9 (1:8)	8 (4:4)	GPL96	22,283	5:12	74	[49]
GSE20164	SN	5 (4:1)	6 (2:4)	GPL96	22,283	6:5	82	[50]
GSE49036	SN	8 (5:3)	15 (7:8)	GPL570	54,675	12:11	78	[51]
GSE43490	SN	5 (2:3)	8 (0:8)	GPL6480	41,108	2:11	74	[52]
GSE42966	SN	6 (2:4)	9 (4:5)	GPL4133	45,220	6:9	73	[53]

## Data Availability

All analyses were conducted in R version 3.6.3 on a laptop (IntelR CoreTM i7-7700HQ CPU 2.80GHz, 8 Gb RAM) running Linux Ubuntu 18.04. All code is freely available online at https://github.com/f-ferraro/CytoarchitecturePDsn (accessed on 11 January 2021). Datasets analyzed can be freely downloaded from https://www.ncbi.nlm.nih.gov/geo/ (accessed on 11 January 2021) using the accession codes GSE7621, GSE8397, GSE20333, GSE20292, GSE20163, GSE20164, GSE49036, GSE43490, GSE42966, and GSE140231.

## Supplementary Materials

The following supporting information can be downloaded at: https://www.mdpi.com/article/10.3390/cells11020198/s1. Supplementary Figure S1: (**A**) Spearman’s correlation between the cell estimates across the studies. (**B**) Violin plots showing the level of expression of representative genes in the microarrays before and after correcting for gender only (original model) in red, or for gender and the cell estimates (corrected model) in blue. CHD9 (cell-unaware LMM β 0.229 ± 0.047 SD BHp 0.0002; cell-aware LMM β 0156 ± 0.053 SD BHp 0.117), and CLK4 (cell-unaware LMM β 0.184 ± 0.043 SD BHp 0.001; cell-aware LMM β 0.107 ± 0.046 SD BHp 0.251), two DEGs of the cell-unaware LMM only; OXTR (cell-unaware-LMM β −0.174 ± 0.075 SD BHp 0.097; cell-aware LMM β −0.32 ± 0.076 SD BHp 0.013) and SYN2 (cell-unaware LMM β −0.023 ± 0.056 SD BHp 0.839; cell-aware LMM β 0.214 ± 0.046 SD BHp 0.005), two DEGs of the cell-aware LMM only. (**C**) Heatmap showing the expression of the DEGs from the cell proportion-aware LMM in the cell types identified in GSE140231. Gene expression values are scaled for visualization purposes; Supplementary Figure S2: Pearson’s correlation matrices of the expression of the top 20 selected markers per microarray dataset from the BRETIGEA package and the human substantia nigra snRNAseq (GSE140231); Supplementary Table S1: BRETIGEA-derived markers for each of the studies analyzed. EWCE results for the BRETIGEA-derived markers in the GSE140231 summary expression matrix; Supplementary Table S2. Sheet 1: Results of the linear model to test the differences in the estimates of the cell types between CTRL and PD. Sheet 2: Results of the metafor meta-analysis of the cell estimates across the studies; Supplementary Table S3: DEGs identified with the cell proportion-unaware and the cell proportion-aware model; Supplementary Table S4: Results of the EWCE of the upregulated and downregulated genes from the cell proportion-aware LMM in the GSE140231 summary expression matrix; Supplementary Table S5: GSEA of the genes ordered by the signed p-values from the two LMMs. Sheet 1: GOCC. Sheet 2: GOMF. Sheet 3: GOBP. Sheet 4: Canonical pathways collection; Supplementary Table S6: Network metrics. Node betweenness centrality, degree centrality, and ranking by the two metrics. Hubs, bottlenecks, and central protein lists; Supplementary Table S7: Results of EWCE for the nodes in the network and the central proteins; Supplementary Table S8: PPI GSEA results. Sheet 1: GOCC. Sheet 2: GOMF. Sheet 3: GOBP. Sheet 4: Canonical pathways collection.
