# Supplementary material for: Correcting Differential Gene Expression Analysis for Cyto—Architectural Alterations in Substantia Nigra of Parkinson’s Disease Patients Reveals Known and Potential Novel Disease—Associated Genes and Pathways
Source: Cells. 2022 Jan 7;11(2):198. doi: 10.3390/cells11020198 (PMC8774027; doi:10.3390/cells11020198)
Supplement: Supplementary file 1 [file cells-11-00198-s001.zip › cells-1543109-SI sent to xml/CorrectingForCellPropPDsn_SupplementaryMethods.pdf]

“Correcting differential gene expression analysis for cyto-architectural alterations in substantia nigra of Parkinson’s disease patients reveals known and potential novel disease-associated genes and pathways”

Supplementary method: Marker sets selection

We considered six brain cell types: astrocytes, endothelial cells, neurons, microglia, ODCs and OPCs. As cell markers, we initially tested 1) the set of markers identified from the substantia nigra single nuclei data (GSE140231), and 2) the set of 5,500 markers obtained from the Brain Cell Type Specific Gene Expression Analysis package version 1.0.0 (BRETIGEA) [19]. To define which of the two sets, the number, and identity of the subsets of markers to employ, we adopted a quality-based heuristic selection. For each marker set, each cell type and study, we ranked the markers on the basis of their association to the 1st principal component of their expression. Next, we calculated the Pearson's correlation among the top 100, and iteratively excluded those showing negative correlations with the majority of the others until no markers with opposite correlations were left. Finally, we selected the top 20 markers for each cell type. We decided to use the BRETIGEA-derived markers in the rest of the analyses since they resolved better into clusters in the correlation matrices (Supplementary Figure S2).
