# Supplementary figures and images for: Correcting Differential Gene Expression Analysis for Cyto—Architectural Alterations in Substantia Nigra of Parkinson’s Disease Patients Reveals Known and Potential Novel Disease—Associated Genes and Pathways
Source: Cells. 2022 Jan 7;11(2):198. doi: 10.3390/cells11020198 (PMC8774027; doi:10.3390/cells11020198)

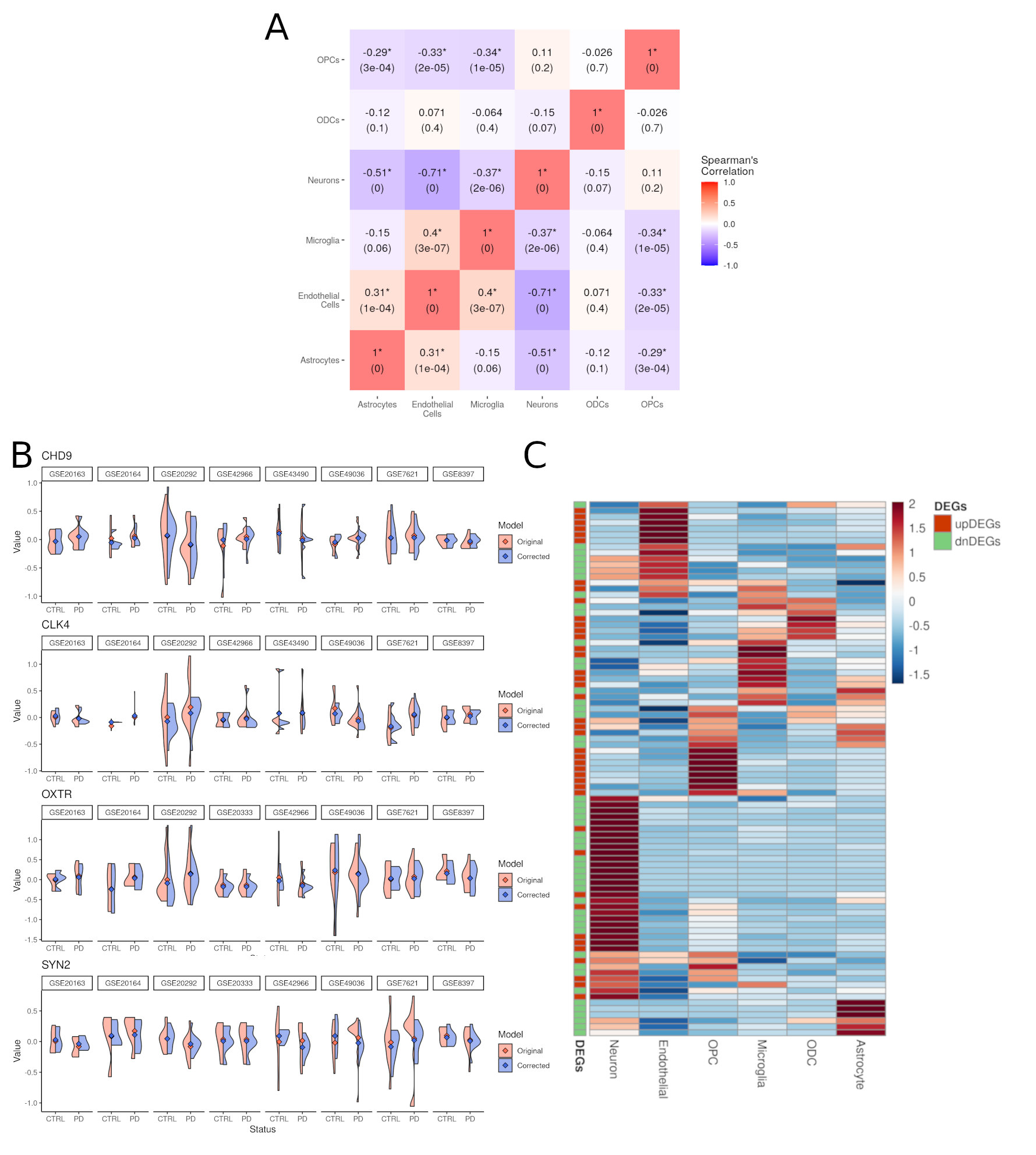

Supplement: Supplementary file 1 [file cells-11-00198-s001.zip › cells-1543109-SI sent to xml/CorrectingForCellPropPDsn_SuppFigS1.tiff]

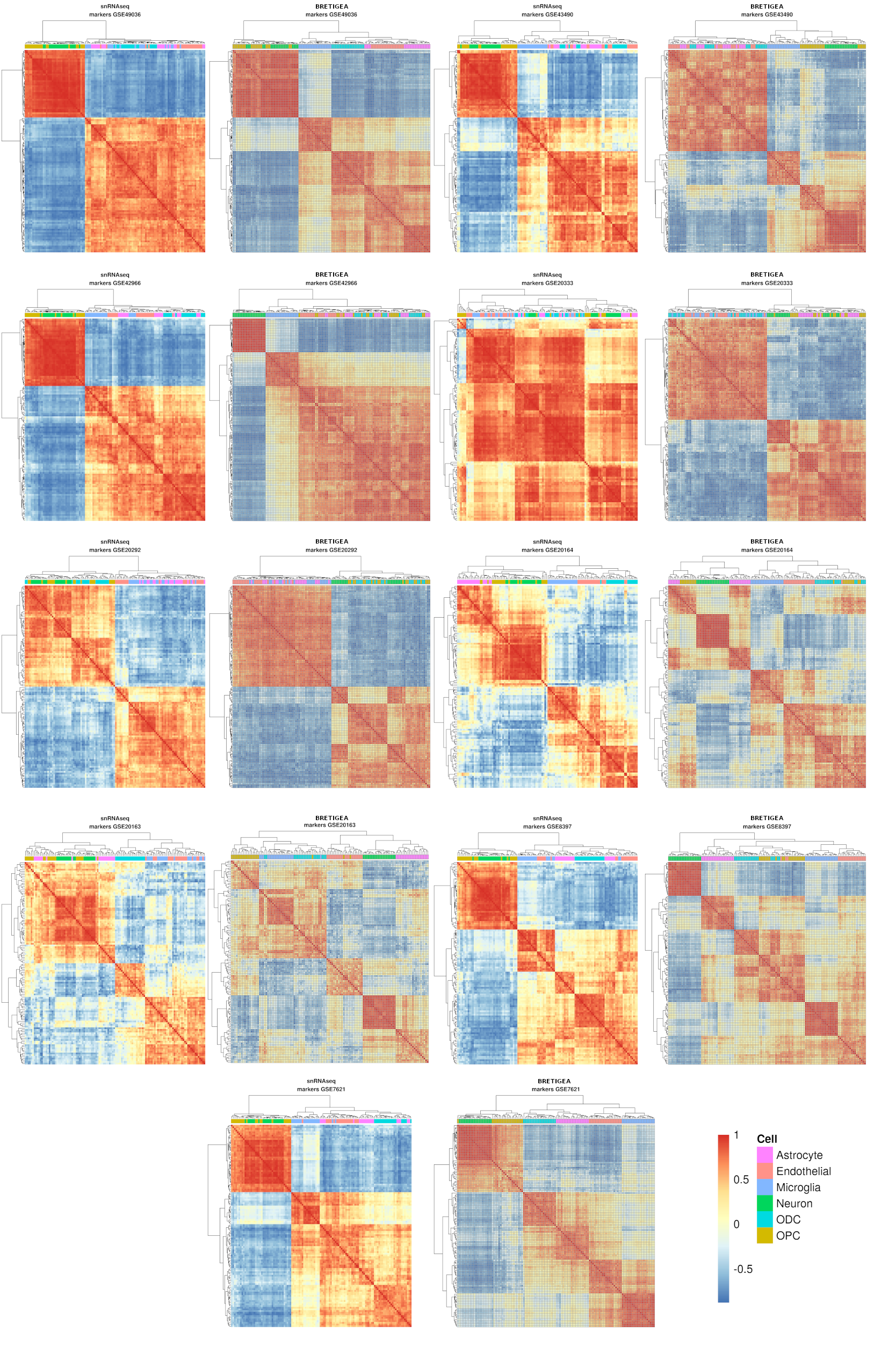

Supplement: Supplementary file 1 [file cells-11-00198-s001.zip › cells-1543109-SI sent to xml/CorrectingForCellPropPDsn_SuppFigS2.tiff]
